# Microscopy analysis of Zika virus morphogenesis in mammalian cells

**DOI:** 10.1038/s41598-020-65409-y

**Published:** 2020-05-20

**Authors:** Lucio Ayres Caldas, Renata Campos Azevedo, Jerson Lima da Silva, Wanderley de Souza

**Affiliations:** 1 Laboratório de Ultraestrutura Celular Hertha Meyer, Instituto de Biofísica Carlos Chagas Filho, Universidade Federal do Rio de Janeiro. Av. Carlos Chagas Filho 373, Prédio CCS, Bloco C, subsolo, CEP:21941902, Cidade Universitária, Rio de Janeiro, RJ Brazil; 2 Instituto Nacional de Ciência e Tecnologia de Biologia Estrutural e Bioimagem. Avenida Carlos Chagas Filho 373. Centro de Ciências da Saúde (CCS), Bloco M, Unidade 3, Cidade Universitária, CEP:21941902, Rio de Janeiro, RJ Brazil; 3 Laboratório de Interação Vírus-Célula, Instituto de Microbiologia Professor Paulo de Góes, Universidade Federal do Rio de Janeiro. Av. Carlos Chagas Filho 373, Prédio CCS, Bloco I, subsolo, CEP:21941902, Cidade Universitária, Rio de Janeiro, RJ Brazil; 4 Instituto de Bioquímica Médica Leopoldo de Meis, Laboratório de Termodinâmica de Proteínas e Estruturas Virais Gregório Weber, Universidade Federal do Rio de Janeiro. Av. Carlos Chagas Filho 373, Prédio CCS, Bloco E, sala 10, Cidade Universitária, CEP:21941902, Rio de Janeiro, RJ Brazil

**Keywords:** Infection, Super-resolution microscopy

## Abstract

Zika virus (ZIKV) is an arbovirus that recently emerged in the Americas as an important pathogen mainly because of its expanded pathogenesis, and elevated tropism for neuronal cells, transposition across the placental barrier, and replication in reproductive tract cells. Thus, transmission modes are eventually independent of an invertebrate vector, which is an atypical behavior for the flavivirus genus and indicates the need to study the replication of this virus in different cell types. Although ZIKV became a target for public health programs, the interaction of this flavivirus with the infected cell is still poorly understood. Herein, we analyzed the main stages of virus morphogenesis in mammalian cells, from establishment of the viroplasm-like zone to viral release from infected cells, using super-resolution fluorescence microscopy and electron microscopy. In addition, we compared this with other host cell types and other members of the *Flaviviridae* family that present a similar dynamic.

## Introduction

Discovered in a sentinel Rhesus monkey of the Zika forest in Uganda in 1947, Zika virus (ZIKV) was isolated for the first time from mosquitoes 1 year later^1^. This previously underestimated arbovirus recently reached the front pages of the main newspapers throughout the world as an international public health emergency. The reason was because of the dramatic increase in outbreaks in the Americas and because of the neurological complications resulting from an infection with ZIKV. The risk of non-vector-mediated transmission, e.g. vertical transmission in humans^2^, blood transfusion^3^, and sexual transmission^4,5^ even from vasectomized men^6^, were highlighted. ZIKV infection during pregnancy was especially discussed because it resulted in morbidity for the fetus. The increase in notifiable cases of microcephaly^7,8^ and Guillain–Barré syndrome^9^ were both shown to be associated to ZIKV infection. Additionally, Joguet *et al*.^5^ recently isolated infectious virus from spermatozoa and showed that ZIKV infection can cause alterations in semen characteristics.

Before the marked decrease in ZIKV infection in the year of 2017 (probably because of herd immunity), the peak periods in the Americas in 2016 resulted in a seroprevalence of 63% in the inhabitants of Salvador, Brazil^10^.

As a member of the *Flaviviridae* family and the *Flavivirus* genus, which also includes the arboviruses dengue (DENV) and yellow fever (YFV), ZIKV harbors seven non-structural (NS1, NS2A, NS2B, NS3, NS4A, NS4B, and NS5) and three structural (capsid, membrane [M], and envelope [E]) proteins^11^.

Internalization is typically mediated by clathrin assistance followed by viral exposure to an acidic environment within the endosomal compartment, which triggers the envelope glycoprotein conformational changes that lead to membrane fusion between the virus and the endosome. The viral (+)RNA is immediately recognized and processed by ribosomes at the endoplasmic reticulum (ER), which originates a precursor polyprotein that is subsequently cleaved by cellular and viral proteases^12^.

*Flaviviridae* members’ replication is cytosolic and it typically causes a rearrangement in the ER in the host, allowing the formation of a viroplasm-like structure, or the viral replication organelle (VRO)^13^. Also called “mininuclei” or “nuclear-like organelles”, the VRO dimensions and characteristics led evolutionists to considerate their role in eukaryotic nucleogenesis^14^. Viral non-structural proteins are involved in ER membrane invaginations, where the viral (−)RNA intermediate synthesis is supposed to occur. Then encapsidation of (+)RNA molecules generated by the viral enzymes bud into the ER lumen. Convoluted membranes (CM) and vesicle pockets (VP) are involved in most *Flaviviridae* biogenesis. The first is probably associated with polyprotein maturation, while the latter seems to be a locus of viral genome amplification, harboring dsRNA and viral proteins^13,15,16^. Finally, cisternae containing viral particles are transported towards the plasma membrane limits, while furin enzymes cleave the M glycoprotein, complementing the virion’s maturation^17^ before egress.

However, ZIKV morphogenesis remains controversial. Although it was previously shown to share similarities with the DENV cellular cycle^18^, ZIKV morphogenesis revealed differences from other members of its family. Additionally, the infection presents distinct dynamics in vertebrate and invertebrate cells, e.g., the presence of syncytia, which occurs only in C6/36-infected cells^19^.

In the present research, we used high resolution imaging techniques including fluorescence and electron microscopy (high resolution scanning electron microscopy (SEM) and electron microscopy tomography) to study the ZIKV VRO of the African (prototype MR766) strain, which was recently demonstrated to be the most pathogenic *in vivo* and *in vitro* (reviewed by^20^), in mammalian epithelial cells (Vero and LLCMK2) and in mouse peritoneal macrophages.

## Methods

### Cells and virus

The mammalian cells LLCMK2 (Rhesus monkey kidney), Vero (African green monkey kidney), and peritoneal-derived macrophages were used in our research. Monkeys have been previously used in studies on ZIKV pathogenesis in non-human primates^21^. Because *Aedes aegypti* and *Aedes albopictus* mosquitoes are implicated in ZIKV transmission, we also used the C636 cells that originated from *Aedes albopictus* and that had also been used as an important model for ZIKV morphogenesis studies^19,22^. Mammalian cells were maintained in RPMI or DMEM (at 37 °C), and mosquito cells were maintained at 28 °C in Leibovitz L-15 medium (Gibco, Life Technologies) that was supplemented with 10% fetal bovine serum (FBS; Sigma-Aldrich), 100 U/mL penicillin, and 100 mg/mL streptomycin. ZIKV African strain MR766, which differs in about 10% at the nucleotide levels compared to the Asian lineage^23^, was used in this study. Recent research on Asian and African strains of ZIKV showed no significant differences in virus replication and susceptibility in human dendritic cells^24^.

### Infection assays

Semi-confluent (80%) cells were infected with 0.5 MOI (multiplicity of infection) of ZIKV in free-serum medium. After an absorption period of 1.5 h at 37 °C and 5% CO_2_, fresh medium containing 2% FBS was added. At specific hours post-infection (hpi), when the cytopathic effect (CPE) was detected using light microscopy, cells were processed for fluorescence or electron microscopy.

### Chemicals

ZIKV labelling was performed by serum from an infected patient at a 1:100 dilution, followed by a secondary (anti-human) antibody at a 1:400 dilution conjugated to AlexaFluor 488 or 546. For endoplasmic reticulum labelling, goat anti-PDIA2 antibody was purchased from Sigma-Aldrich Co. For dynamin labelling, primary anti-dynamin antibody (Invitrogen, Carlsbad, CA, USA) was used at a 1:100 dilution and incubated with a 1:400 dilution of the secondary goat anti-mouse IgG (H + L) antibody that was conjugated to AlexaFluor 488 (Invitrogen). Actin filaments were stained with phalloidin red (Sigma-Aldrich) diluted 1:40 in PBS, for 20 min in the dark.

### Super-resolution microscopy

Mammalian or mosquito cells were grown on glass coverslips and fixed in 4% formaldehyde in phosphate buffered saline (PBS), pH 7.2, for 2 h. Next, samples were washed three times with PBS at room temperature, and permeabilized with 0.1% Triton X-100 in PBS for 10 min. During pre-incubation, samples were treated with 50 mM ammonium chloride and 3% BSA in PBS, pH 8.0, for 45 min. The samples were then incubated with primary antibodies for 1 h, rinsed and then incubated with secondary antibodies for 1 h. Finally, samples were incubated with 10 μg/mL 4,6-diamidino-2-phenylindole (DAPI) (Sigma-Aldrich) to allow cell nucleus visualization and the coverslips were mounted in 2.5% 1,4-diazabicyclo (2,2,2)-octane (DABCO) (Sigma-Aldrich) to prevent loss of fluorescence. Images were recorded in a Zeiss Elyra PS.1, using Super Resolution Structured Illumination Microscopy (SR-SIM) mode, using five phases and five rotations that generated an image’s algorithms, which were subsequently solved using ZEISS software ZEN 2012 (version 9.1.1.5).

### En bloc processing for transmission electron microscopy

For transmission electron microscopy (TEM) analysis, mock and infected monolayers in 25 cm^2^ plastic culture flasks were fixed in 2.5% glutaraldehyde in 0.1 M cacodylate buffer (pH 7.2), and post-fixed for 1 h in 1% OsO_4_/0.8% potassium ferrocyanide in the same buffer. Samples were then incubated with 2.5% uranyl acetate in water for 2 h, washed three times in 0.1 M cacodylate buffer (pH 7.2), dehydrated in ethanol and embedded in Polybed resin (Polysciences). Ultrathin sections were stained with 5% uranyl acetate (40 min) and 4% lead citrate (5 min) before observation using a FEI Tecnai T20, a FEI Tecnai G20 or a FEI Tecnai Spirit transmission electron microscope. These microscopes were respectively equipped with the following cameras: Megaview 1 K, Eagle 4 K and a Veleta 2 K. Images ranged in magnification from 20000-200000 times, and pixels varied from 2-10 nm.

### Scanning electron microscopy

For SEM, the infected and mock cells grown over round coverslips in 24-well plates were fixed, post-fixed, and dehydrated as described above. Then, the coverslips were critical-point-dried in CO_2_ in a Balzers CPD apparatus before monolayer scraping with Scotch tape™ and sputtering with a 4-nm thick carbon coat in a Balzers apparatus. Samples were observed using an Auriga ZEISS microscope. Backscattering electrons were also used to optimize the contrast of some features of the infected cells.

### Electron microscopy tomography

Semi-thin sections (200–250 nm) of polybed-embedded samples were collected on 100-mesh copper grids. After staining with uranyl acetate and lead citrate, the grids were incubated with 10 nm colloidal gold for 10 min, allowing the alignment (fiducial markers) of the tomographic series. The tomographic series was acquired as described by Paredes-Santos *et al*.^25^, and tomograms were computed for each tilt axis. Tomogram analysis and modelling were performed in the IMOD software package^26^. Structures of interest were contoured manually in serial slices that were extracted from the tomogram and three-dimensional (3D) models were rendered to investigate the 3D geometry of the selected structures.

## Results

To compare the subcellular reorganization resulting from ZIKV infection in different cells, we infected monkey cells (LLCMK2, Vero) and mouse peritoneal macrophages, as well as mosquito C636 cells with 0.5 MOI of the virus. Previous studies showed that when 15 nonhuman cell lines were screened, LLCMK2 and Vero cells displayed the highest ZIKV viral loads^27^. After the establishment of CPE, where rounding and detached cells were observed, the cells were fixed and processed for microscopic analysis. Our light microscopy analysis of ZIKV-induced CPE is in close agreement with the data from Barreto-Vieira *et al*.^19^, in which Vero cells exhibited a more pronounced detachment than C636 cells (data not shown) because the latter is more adapted; this is also in agreement with *A. albopictus* non-fatal infection rates^28^.

**Figure 1:**
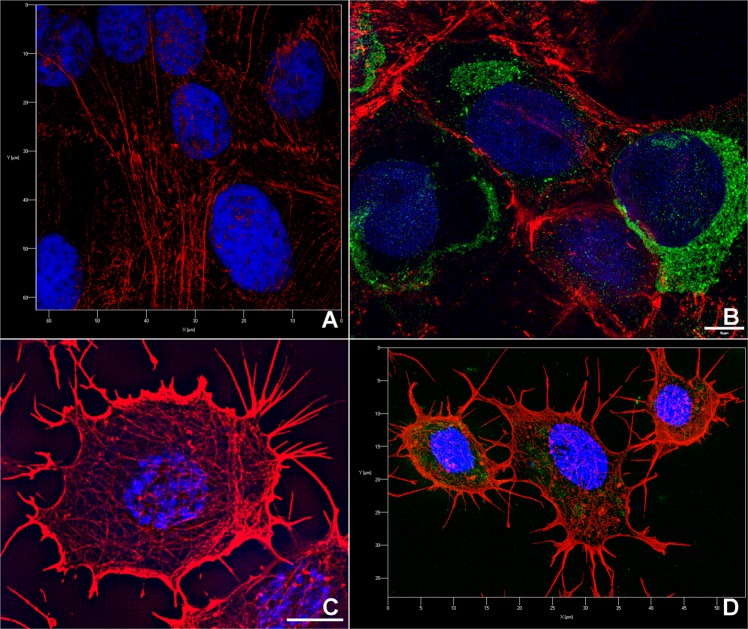
SIM mode of super-resolution microscopy of mammalian and insect cells infected with ZIKV and labelled for indirect immunofluorescence. **(A)** Mock-infected Vero cells; **(B)** ZIKV was detected in the perinuclear region of the cell; **(C)** Mock-infected C636 cells; **(D)** 3D image of C636 cells infected with ZIKV showed labelling for viral proteins and virions that were widely distributed throughout the cytoplasm, and not densely confined around the nucleus. ZIKV is labelled in green, actin is labelled in red, and the nucleus is labelled in DAPI. The samples were fixed at 7 days post-infection (dpi). Bars, **(B,C)** 5 µm.

Indirect immunofluorescence microscopy observed by super-resolution microscopy (Fig. 1) labelled ZIKV predominantly in the perinuclear region of Vero (Fig. 1B), and more spread by the cytoplasm of C636 cells (Fig. 1D) at 96 hpi. This approach also revealed the presence of the virus in dividing mammalian cells 1 week after infection (Supplemental Material 1).

**Figure 2:**
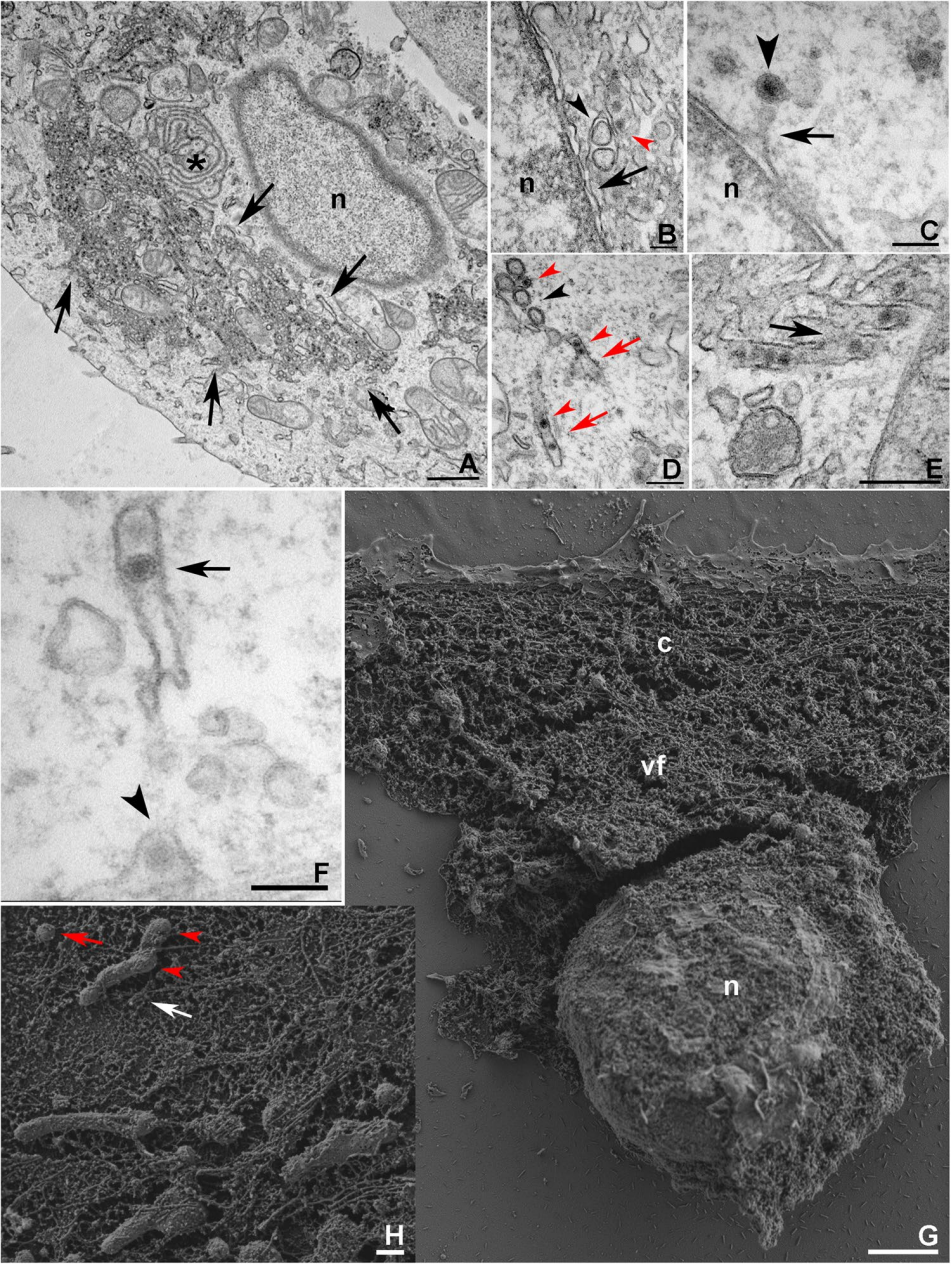
Electron microscopy of the ZIKV replication factory in mammalian cells. TEM analysis of **(A)** ER expansion and modification induced by ZIKV infection in LLCMK2 cells at 7 dpi. The asterisk marks the perinuclear site at which evident ER alterations were clearly observed. The arrows point to the VRO. **(B)** Involvement of the mouse macrophage outer nuclear envelope (black arrow) and vesicle packets (black arrowhead) opposing ER sheets containing the virus (red arrowhead). **(C)** ZIKV particle (arrowhead) near the LLCMK2 outer nuclear envelope (arrow). **(D)** Tubular altered ER in LLCMK2 cells containing immature viral particles were observed in close proximity to the VPs. A presumed step of ZIKV (red arrowheads) morphogenesis occurs by the association of VPs (black arrowhead) and ER sheets that were derived from tubular structures (red arrows). Connections (black arrow) between some of these tubules containing ZIKV were noticed in infected macrophages at 4 dpi **(E)**, while other tubules were apparently closed. Opened (arrowhead) and closed (arrow) forms of this structure could be observed in infected LLCMK2 cells **(F)**. **(G)** Membrane scraping of infected LLCMK2 cells allowed SEM analysis of the cell nucleus (n), cytoplasm (c), and ER reorganization (vf) revealing the closed shape of some tubular ER profiles (white arrow), which probably contain VPs (red arrowheads) and free spheroids (red arrows) **(H)**. Bars, **(A)** 1 µm; **(B,C,F)** 100 nm; **(D,E,H)** 200 nm; **(G)** 2 µm.

The data obtained by fluorescence microscopy supported a more precise investigation that was conducted by TEM analysis of ZIKV morphogenesis in LLCMK2 cells, showing ER expansion and reconfiguration (Fig. 2A). Despite the fact that the same MOI was used for LLCMK2 and mouse peritoneal macrophages infections, the latter exhibited less pronounced alterations in the cytoplasm, and they were shown to originate from the outer nuclear envelope (Fig. 2B). In this Figure, the proximity of two ER sheets housing VP or spherules, and immature virus particles, respectively, suggests budding of the latter to sub-compartments containing the VPs.

Although the nuclei of infected cells examined did not contain ZIKV particles, these were eventually observed to be near the outer nuclear envelope (Fig. 2C). Tubular regions of the altered ER were found to contain immature viral particles (Fig. 2D), and these structures were in close proximity to the VPs. Membrane openings, which suggest a gateway to the nascent viral genome, were observed at the ER derived tubular structures (Fig. 2D). Some of these tubules were shown to be inter-connected (Fig. 2E), while others were apparently closed (Fig. 2F). Rearrangement of the ER in infected cells was also seen using SEM, confirming the closed shape of some tubular ER profiles (Fig. 2G–H).

**Figure 3:**
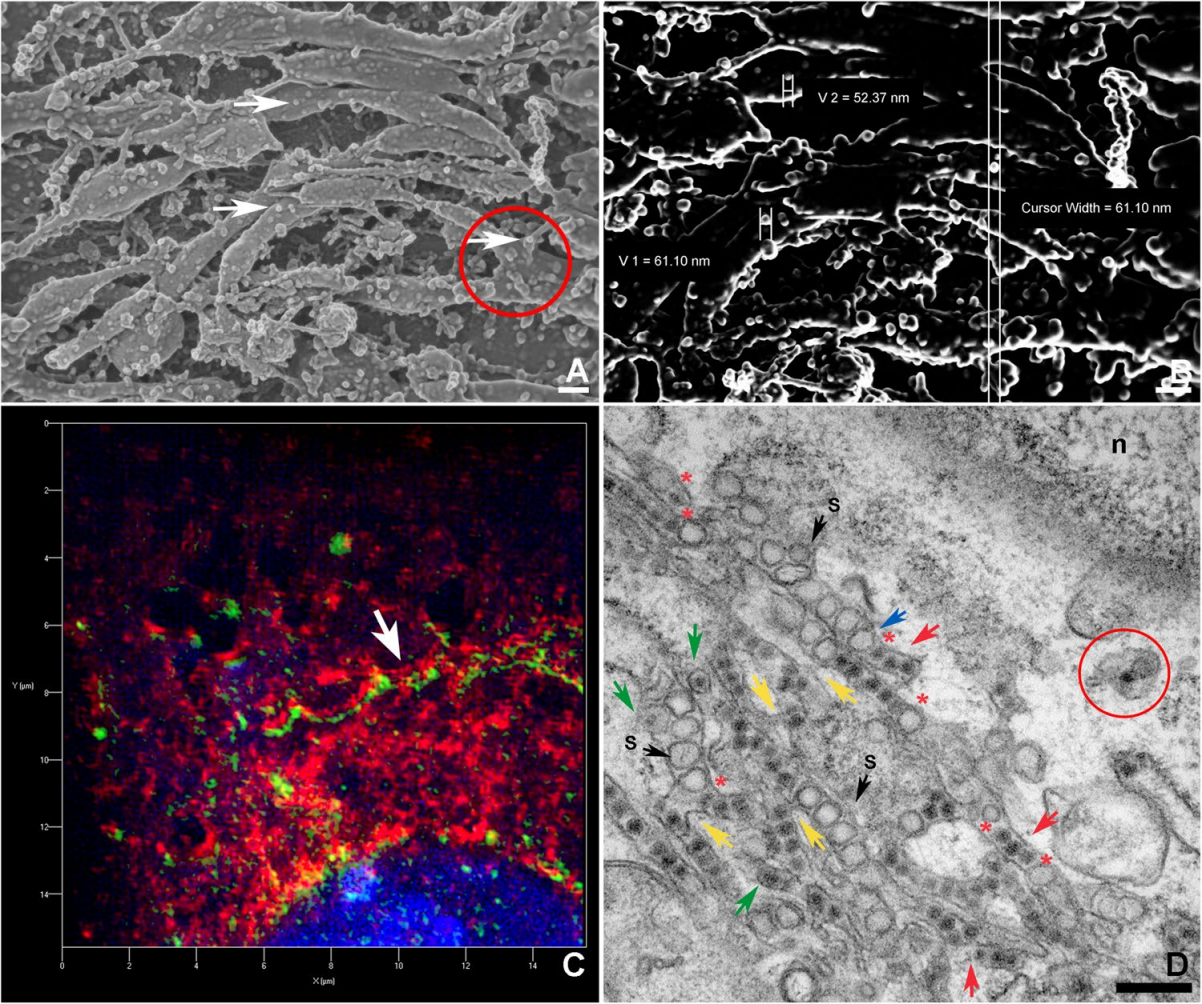
Microscopic analysis of the viral factory details in LLCMK2 cells. **(A)** SEM imaging of the ZIKV VRO tubular compartments revealed spheroid structures on their surface (arrows). One of the connections between these compartments is indicated by the red circle. **(B)** Image of the same region, acquired by backscattering electrons, revealed the diameter (50–60 nm) of the round particles that adhered to the modified ER surface. **(C)** SIM-SR fluorescent labelling for endoplasmic reticulum (red) and ZIKV proteins (green) showed the close association between them and a parallel disposition (arrow). **(D)** Spheroids from different sizes (black arrows, “s”) were observed to be separated from the immature virus particles (red arrows) in distinct compartments. However, connections between both compartments were also seen (yellow arrows). A pore was also observed in some spheroids (blue arrow), and viral particles were found to share the same compartment as the spheroids (red asterisk). The compartments containing spheroids and viral particles eventually displayed viral particles inside the spheroids (green arrows). The red circle highlights the presumed interaction between an immature virus and a membrane that seems to wrap around it. n, nucleus; Bars, **(A,B,D)** 200 nm.

SEM also allowed visualization of the VRO’s details (Fig. 3A). The backscattering electron mode revealed that particles with a diameter ranging from 50 to 60 nm adhered to the surface of the tubular region in the modified ER (Fig. 3B), suggesting the occurrence of a viral assembly locus before internalization and luminal routing. The ER was labelled for SR-SIM fluorescence microscopy, revealing a pattern in which the viral proteins always seemed to be associated with it, eventually displaying a parallel orientation to this organelle (Fig. 3C).

Moreover, this is consistent with TEM observations of the VRO (Fig. 3D). In this Figure, many spheroids resulting from ER membrane invagination showed an average diameter of 80–100 nm and were most observed in different compartments compared to the immature virus particles. However, in some images, these compartments were shown to interconnect themselves and pores connecting spherical vesicles to the cytosol were also observed, allowing viral RNA transit. Immature viral particles were also found within the same tubular structure, but they were in a row opposite of the VPs. Some viral particles were found inside the VPs, suggesting an intermediate and unprecedented form.

**Figure 4:**
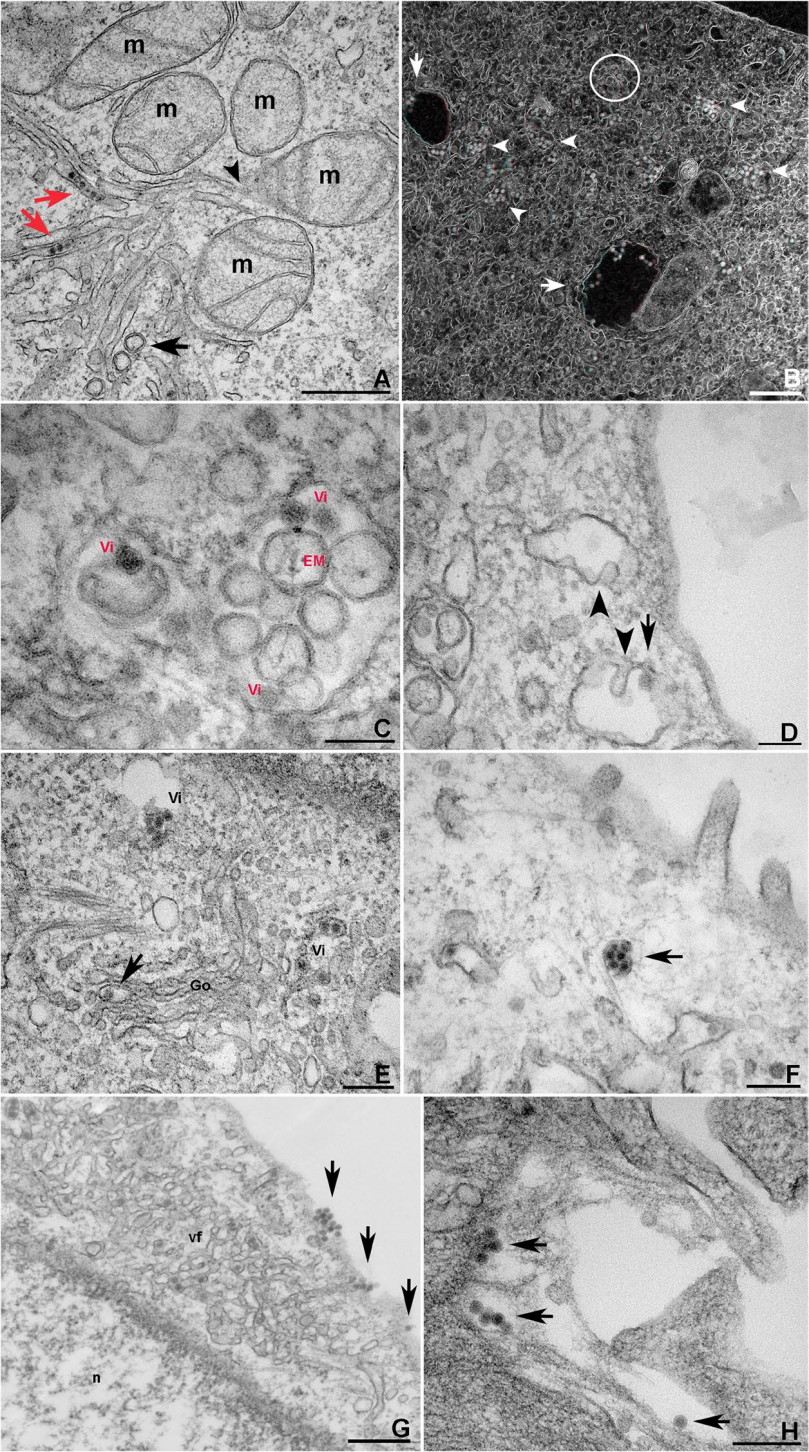
Electron microscopy of ZIKV intracellular trafficking. **(A)** In LLCMK2 cells, ER sheets containing virus (red arrows) and CMs (black arrow) converged into a zone where mitochondria (m) is abundant. The black arrowhead shows the apparent association between the ER sheets and mitochondria. **(B)** In the same cell type, vacuoles (arrows) and cisternae (arrowheads) containing virus are shown by STEM (use red and green glasses). Loci of ER sheets containing virus and compartments containing spheroids were also detected (circle). **(C)** Virions (Vi) and spherical membranes (EM) were shown to interact inside the vacuoles. Membrane invagination observed in mouse macrophages **(D)** results in the internalization of empty spheroidal membranes (arrowheads), and, at a lower rate, virions (arrow), which acquire the viral envelope. **(E)** The previous virus maturation step was detected in the Golgi apparatus (Go). The arrow indicates the transit of one viral particle, while other virus particles (Vi) were found near the organelle. **(F)** The transit of virions towards the LLCMK2 cell plasma membrane was shown to also occur within tight cisternae (arrow). **(G,H)** The fusion of cisternae or virus-containing vacuoles with the cell plasma membrane results in the release of groups of virus by exocytosis, which is suggested to occur (arrows), in mouse macrophages at 4 and 5 dpi respectively. Bars: **(A,B,G)** 500 nm; **(C,D)** 100 nm; **(E,F,H)** 200 nm.

The same arrangement was observed near the mitochondria, where VPs and immature virus appeared on opposite poles (Fig. 4A). In this Figure, spherule-containing ER profiles were seen bordering the ER membrane leaflets. An apparent association between mitochondria and the ER compartments was also observed. The viral particles were shown to accumulate in cisternae and vacuoles (Fig. 4B) before translocation to the host cell plasma membrane. Some large vesicles contained empty small vesicles, while others also contained virus particles. However, vacuoles containing both were also observed (Fig. 4C). These small vesicles resulted from the large vesicle’s invagination, and viral particles are probably internalized after membrane fusion of virus-containing CM with these large vesicles (Fig. 4D). In this step, ZIKV appears to acquire its envelope from these membranes.

**Figure 5:**
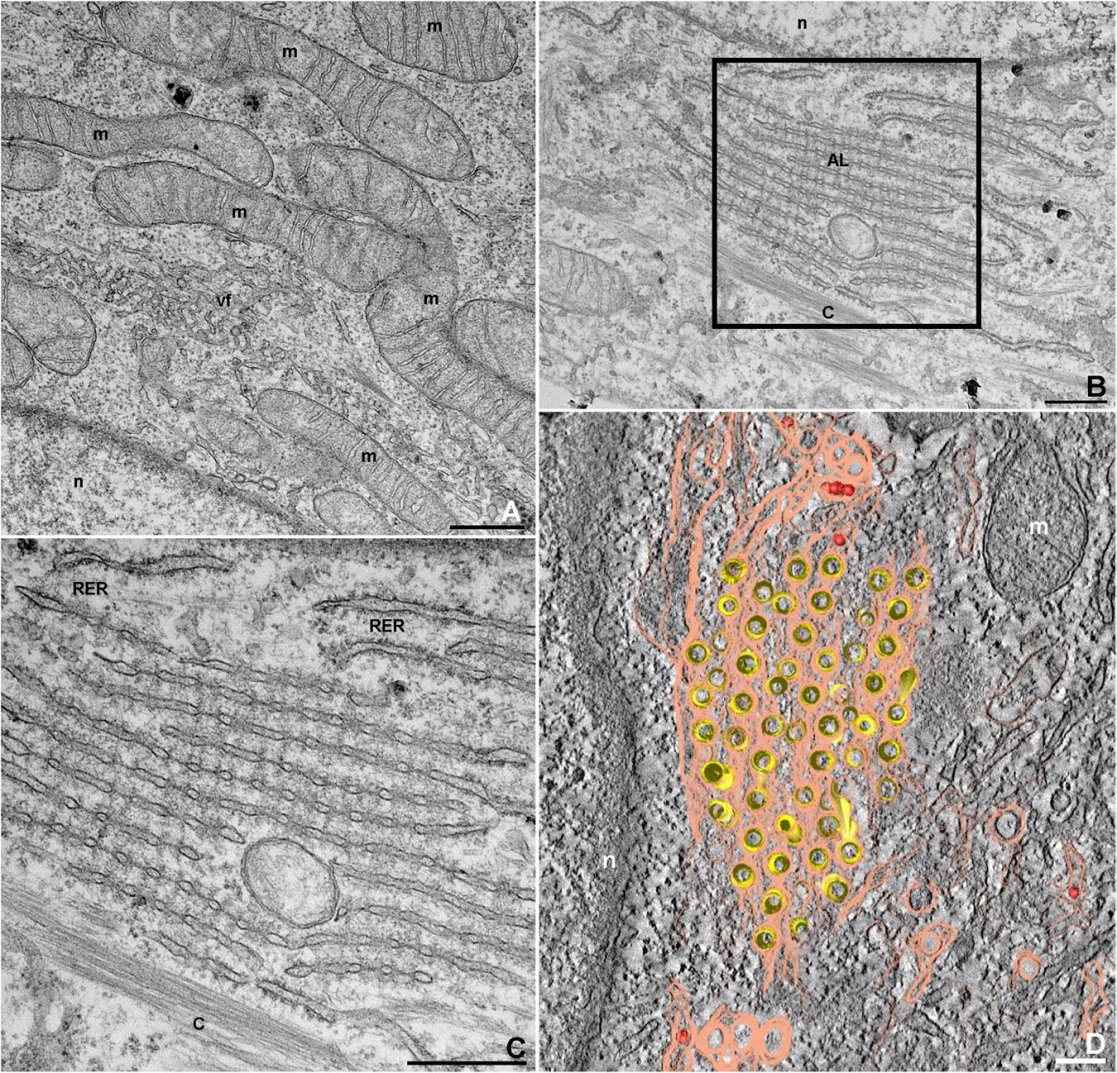
Electron microscopy of mitochondria and annulated lamellae during ZIKV infection in mouse macrophages. **(A)** Clusters of elongated mitochondria (m) around the viral replication organelle (vf) in mouse macrophages (n, nucleus). **(B)** Actin-like filaments (c) were detected near perinuclear (n, nucleus) annulated lamellae (AL). The selected area is shown in **(C)**, where AL seem to display a continuity with the RER. **(D)** Tomogram of perinuclear annulated lamellae with ZIKV particles depicted in red. (m) mitochondria; (n) nucleus; Bars: **(A**–**C)** 500 nm; **(D)** 100 nm.

Viral processing of immature virions involves conformational changes caused by acidic environments across the Golgi, where a host furin-like protease cleaves the viral prM (17). The number of viral particles in transit across the Golgi or around this organelle was lower when compared to the rest of the cytoplasm region. Electron microscopy of this step revealed opposing CMs and virus-containing cisterns (Fig. 4E), and the latter carried the virions towards the host cell plasma membrane (Fig. 4F), culminating in their content release into the extracellular medium (Fig. 4G–H). Clusters of elongated mitochondria were observed in the perinuclear sites of ZIKV-infected cells (Fig. 5A).

Annulated lamellae (AL) were present in several infected macrophages (Fig. 5B–D), in the perinuclear region (Fig. 5B) and near the cell periphery (data not shown). The association of ER with this structure is suggested in Fig. 5C. Elongated mitochondria were also observed at these sites (data not shown). In Supplemental Material 2, tomography of the AL revealed ZIKV particles wrapped in membranes that are shared with this organelle (Fig. 5D).

## Discussion

### ZIKV morphogenesis

Infections of different cell models with distinct ZIKV strains were recently studied in detail^29,30^. This work aims to provide more cellular biology details of the interaction between ZIKV MR766 and three types of mammalian cells.

Generally, modification of the eukaryotic endomembrane system caused by some flavivirus infections results in viral replication that is confined to a locus, which concentrates cellular and viral factors that are crucial for virus replication. This compartmentalization also leads to task segmentation within this replication process, avoiding contact of innate immune sensors with the genomic RNA^12^.

The ER displays a network of membranous sheet-like reservoirs and tubes^31^ that spread from the cell nucleus. The close association of ZIKV morphogenesis with these endoplasmic reticulum-derived membranes suggests that transport of the nascent virions occurs through the secretory pathway towards the host cell plasma membrane, from which they acquire their cell-derived lipid bilayer that encapsulates the C-protein^32^. Recent data from Offerdahl *et al*.^29^ showed that ZIKV E glycoprotein and dsRNA (indicating viral replication) overlapped with the PDI staining in neuroblastoma cells. Our protein disulfide isomerase (PDI) labelling of the ER showed co-localization with ZIKV, and the pattern of this labelling is consistent with our TEM observations (Fig. 3C,D). This was significantly different from the PDI distribution compared to double-stranded RNA in ZIKV MR766-infected Huh7 cells^30^.

Our findings of multiple spherules or virus-induced vesicles within the tubular sites of the modified ER as well as accumulation of CM around this cellular organelle are consistent with previous studies on ZIKV Puerto Rico strain PRVABC59 interactions with Vero E6 cells^33^ and ZIKV MR766-infected Huh7 cells^30^. Moreover, the electron-dense content that supplies the VP shown by our *in bloc* sample processing, supports the hypothesis of viral genome accumulation in these structures. Replication of DENV is also presumed to occur at these sites, as shown by the presence of double-stranded RNA^13^. Unlike West Nile virus^34^, there were no interconnections between spherules within the modified ER.

Because virion budding within ER cisternae occurs in DENV-infected cells, which is the opposite of invaginated vesicle pores, the same results were expected for ZIKV-infected cells, as suggested in Figs. 2B,D and 4A. However, our data show that VP and the encapsidated viral particles dominantly shared the same ER tubular structure (Fig. 3D). The double-membrane vesicles (spherules), which are commonly observed in other replicating flaviviruses^35^, appear to share the same sub-compartment for ZIKV. This is consistent with the recent findings for the Puerto Rico strain, which suggest that the invagination of immature viral particles into the ER membranes occurs before packaging within containing spherules, leading to an assembly process that culminates in discarding the spherule membrane. According to this model, empty spherules correspond to inactive viral transcription sites^33^. Once processed within these spherules, the assembled but immature viral particle would eliminate these membranes, appearing free in the ER lumen when observed by electron microscopy.

The sites of the modified ER through which the viral transcription intermediates and the immature virus may transit was, for the first time, documented by SEM, and backscattering electrons contributed to measuring the virus-like structures on the ER surface (Fig. 3A,B). The assessed dimensions are consistent with the assembling virions and they are also incompatible with the ribosome size. Recently, Arakawa & Morita^36^ hypothesized similar structures which are supposed to contribute to the virus assembly when reaching the lumen of the spherules, possibly corresponding to the newly synthesized RNA-containing capsid, about to sprout into the ER or coat protein complexes harboring progeny virus particles in their route to a secretion pathway.

Since we observed the modified ER using transmission and scanning electron microscopy, these approaches could reveal two distinct shapes for this organelle. The structures reported in Fig. 3A,B were not found in Fig. 3D (TEM) and 2H (SEM). Intriguingly, this could point to the need for further investigation regarding different specialized loci of modified ER during virus processing.

The cisternae-like compartment, which results from a dilated ER site and seems to harbor these particles in their vicinity (Fig. 4B), is thought to meet the Golgi apparatus, leading to the maturation stage. Consistent with data from the ZIKV Puerto Rico strain^33^, the ZIKV African strain MR766 was also observed in individual transit through the Golgi (Fig. 4E). In this step, host cell furin cleaves the precursor prM protein, resulting in the small M protein and the pr fragment. The latter leaves the viral particle during the virus exit. We observed similar cisternae nearby the cell plasma membrane (Fig. 4F).

Although studies have shown the occurrence of paracrystalline array of ZIKV nucleocapsids within the ER cisterns of Vero cells^19^, this was not observed in any of our models (considering the type of cells and virus strain used). Besides that, we also did not observe other viroplasm-like structure different from that established in the ER region.

The Zippered rough ER was previously defined as tightly juxtaposed and collapsed ER cisternae^30^, and it was supposed to represent a transition between sub-compartments that restrict the viral particle transit. However, this was not observed in the processed samples from our cell-virus models.

### Virus-induced vacuolization

Generally, infections with viruses from the *Flaviviridae* family (e.g. DENV) can cause significant reorganization of the host cell secretory pathway. Recent studies using Hela cells and primary human astrocytes infected with ZIKV strains HD78788, PF13, and NC14 showed the induction of vacuoles that originated from ER membranes^37^. These studies also suggested that the borders of these vacuoles could serve as an intermediate locus for viral replication because of the viral RNA and E protein concentrations. However, the antigenic-related virus BVDV (*Flaviviridae* family, *Pestivirus* genus) also induces vacuoles, although this seems not to be crucial for its replication^38^. This phenomenon of vacuolization, however, was not detected in our cell models, where virus-containing cisternae were present. While vacuole formation and cell death were previously indicated as promoters of ZIKV release and spread^37^, it seems plausible that, in our model, the virus-containing cisternae could perform virion exocytosis.

Although myelin figures were abundant in Vero cells infected with ZIKV^18^, these structures were not numerous during our observations in ZIKV-infected LLCMK2 and macrophages (Fig. 4B). However, multivesicular bodies and multilamellar myelin-like structures may not be specific to ZIKV infection^33^.

### CMs and mitochondria alterations

The absence of CMs was previously reported in DENV-infected mosquito cells^39^. Although the role of CM remains unclear, studies on Kunjin virus morphogenesis suggest that these structures are highly dynamic sites of polyprotein maturation, and they were subjected to merging and parting in recent studies on DENV infection^15,40^. Moreover, CM structures could interfere with mitochondrial antiviral (MAV) platforms by avoiding DRP1 translocation and mitochondrion fission^40^.

Viral synthesis within the viroplasm-like structure requires a large amount of energy, and it is conceivable that mitochondria may be present on their periphery. Cortese *et al*.^30^ showed peripheral localization of mitochondrion in ZIKV-infected human neural progenitor cells (hNPC). However, in LLCMK2, for example, the cell organelles were concentrated at the perinuclear sites as well as among the interface between CMs and the tubular viroplasm structures in the infected cells. Kim *et al*.^41^ observed the same pattern with hepatitis C virus (HCV) infection and in mitophagosomes. Our data could help their hypothesis that this phenomenon is associated with ATP pools at viral replication sites. However, the elongated shape of the mitochondria in macrophages infected with ZIKV is in agreement with the morphology observed in Huh7 cells that are infected with DENV^40^. In that case, mitochondrial elongation results from inactivation of the fission factor dynamin-related protein 1 (DRP1) by the viral protein NS4B. Our data showed that mitochondrial elongation was also noticed at regions where the CMs converged with the tubular ER that contained the virus (Fig. 4A).

The replication of other (+)RNA viruses, such as the Flock House virus, is closely associated with the mitochondria. The RNA replication of this nodavirus occurs within spherules that invaginate from the outer mitochondrial membrane^42,43^. We could not detect a similar pattern in any of the cell lines that we used in our work. However, in Fig. 2A, the presence of mitochondria is notable in the area of the altered ER. This proximity is reinforced in Figs. 4A and 5A, where the mitochondria and ER seem to be associated.

### Nucleus

Following previous research^44^ that showed the participation of the cell nucleus in the flavivirus morphogenesis, Buckley and Gould^45^ reported the observation of a ZIKV envelope protein within VERO cell nuclei using indirect immunofluorescence. This could reflect an early step in the ZIKV infectious cycle. In our study, however, we could not detect ZIKV proteins at this site using fluorescence or electron microscopy. However, neither Buckley and Gould^45^ nor our group could detect these presumed nuclear viral particles using electron microscopy.

In contrast to the kidney-shaped form assumed by ZIKV MR766-infected Huh7 cells^30^, no alterations in the nuclear morphology of Vero, LLCMK2, or macrophage infected cells were seen using fluorescence microscopy, or using TEM or SEM (Figs. 1B and 2A,G).

### Annulate lamellae

Annulate lamellae (AL) can occur in almost all types of cell, and they are described as cytoplasmic organelles that contain pore complexes, which are organized in a symmetrical form and are presumably involved in nuclear transport. This organelle could act as a locus for import complex assembly and export complex disassembly. Besides high cell proliferation conditions, cell-cycle arrest is also related to the abundance of AL. Independently, viral infections or chemical treatments can increase or induce its formation^46^.

Although the AL depicted in Fig. 5B–D were similar to the CMs that were crossed by microtubules that are observed in ZIKV MR766-infected Huh7 cells^30^, previous studies have shown the occurrence of this organelle in HSB-2 cells infected with human herpesvirus type 6, where the organelle formation coincided with the expression of the viral glycoprotein gp116^47^. In that study, the AL pore complexes appeared to be in continuity with rough endoplasmic reticulum membranes, and we also noticed this in ZIKV-infected macrophages (Fig. 5C). The occasional appearance of this organelle in fetal Rhesus kidney cells infected with the hepatitis A virus also suggested a role in virus morphogenesis^48^. Taking into account that the viral infection can alter host cell protein synthesis, establish new priorities, and eventually lead to its arrest, it is reasonable that AL is introduced. However, because we did not notice viral particles within the AL in our virus–cell system, the hypothesis of immature ZIKV storage was not supported.

In conclusion, the present data contribute to the resolution of the intricate puzzle characterized by the ZIKV morphogenesis. Among the main points of this work we would like to highlight (1) the infection of dividing mammalian cells; (2) the confirmation of closed profiles of tubular compartments that harbor viral particles by SEM imaging; (3) the panoramic structure of the VRO imaged by SEM; (4) the details of the locus of viral processing in the modified ER by the using of BSE; (5) the presence of VP and encapsidated viral particles within the same ER tubular structure, and; (6) the presence of AL in infected macrophages.

Flaviviruses are presumed to display a conserved morphogenesis mechanism. However, ZIKV is the first flavivirus that is involved in sexual transmission in humans, and vertical transmission leads to congenital abnormalities. Moreover, the existence of these different routes of infection presupposes a distinct kind of viral morphogenesis, or a myriad of cells harboring ZIKV. Because of the existing gap in understanding ZIKV pathogenesis, investigating its interaction with the host cell at the cell biology level is crucial for designing new lines of disease containment. Herein, we approached the main changes that occur in ZIKV-infected mammalian cells to contribute to a better understanding this morphogenesis. However, efficient infection in cells of other mammalian and avian species need further investigation because of their potential role in ZIKV epidemiology.

## Supplementary information

Supplementary Video.

Supplementary Video

## Data Availability

The data that support the findings of this study are available from the corresponding author on reasonable request.
